# A prospective observational study to assess PD-L1 expression in small biopsy samples for non-small-cell lung cancer

**DOI:** 10.1186/s12885-019-5773-3

**Published:** 2019-06-07

**Authors:** Akihito Tsunoda, Kei Morikawa, Takeo Inoue, Teruomi Miyazawa, Masahiro Hoshikawa, Masayuki Takagi, Masamichi Mineshita

**Affiliations:** 1 Division of Respiratory Diseases, Department of Internal Medicine, St. Marianna University School of Medicine, 2-16-1 Sugao, Miyamae-ku, Kawasaki, 216-8511 Japan; 2 Department of Pathological Diagnosis, St. Marianna University School of Medicine, 2-16-1 Sugao, Miyamae-ku, Kawasaki, 216-8511 Japan

**Keywords:** Lung cancer, Bronchoscopy, Pathology, Immunohistochemistry

## Abstract

**Background:**

Programmed cell death-1 (PD-1) immune checkpoint inhibitor antibody has proven to be effective in advanced non-small cell lung cancer (NSCLC) patients positive for programmed cell death-1 ligand-1 (PD-L1). However, there are currently no prospective studies evaluating PD-L1 expression for small biopsy samples.

**Methods:**

To prospectively investigate the reliability of small samples for NSCLC, we included patients who underwent diagnostic biopsy by flexible bronchoscopy, computed tomography (CT) and ultra-sonography (US) guided core-needle to determine the PD-L1 expression status. In pathologically confirmed NSCLC, PD-L1 expression was evaluated using companion diagnostic PD-L1 immunohistochemistry. We evaluated: 1) tumor cell count and sample size, 2) tumor proportion score (TPS): <1, 1–49%, 50%≦, and 3) the concordance rate of TPS by biopsy and surgical samples.

**Results:**

Of the 153 cases of PD-L1 expression, 110 were assessed using endobronchial ultrasonography guided transbronchial biopsy (EBUS-TBB) (thin bronchoscopy 84 cases; normal bronchoscopy 26 cases), 23 were endobronchial ultrasonography guided transbronchial needle aspiration (EBUS-TBNA), and 20 cases of CT or US-guided core-needle biopsy. Tumor cell count and sample size were significantly larger for normal bronchoscopy than thin bronchoscopy or EBUS-TBNA samples. Moreover, tumor cell counts for each subsequent biopsy decreased. In all cases, TPS distribution (undiagnosed, <1%, 1–49, 50%≦) was 2.6, 34.6, 31.4, 31.4%, respectively. TPS positive cases using thin bronchoscope was 55.9%, normal bronchoscope was 73.1% and EBUS-TBNA was 78.3%. In early stage adenocarcinoma, TPS was lower compared with advanced stages. Conversely, in squamous cell carcinoma, the rates of TPS were similar regardless of stage. The concordance rate of TPS by biopsy and surgical materials was 86.7%.

**Conclusion:**

Utilizing smaller samples for evaluation, the frequency of TPS was comparable to past clinical trials using larger samples. The differences in TPS were influenced by diagnostic tools, cancer histologic types and staging. The concordance of TPS between EBUS-TBB samples and surgical materials was high.

**Trial registration:**

This study was performed at the Department of Respiratory Medicine at St. Marianna University School of Medicine Hospital, with ethics approval (#3590) and registered as a clinical trial (UMIN000027030).

**Electronic supplementary material:**

The online version of this article (10.1186/s12885-019-5773-3) contains supplementary material, which is available to authorized users.

## Background

The development of immune checkpoint inhibitors has changed chemotherapy for non-small cell lung cancer (NSCLC) and other malignancies. In recent years, many immune checkpoint inhibitors were developed and approved after promising results in clinical trials. In Japan, nivolumab, pembrolizumab, and atezolizumab are approved for the treatment of advanced NSCLC [1–5].

Pembrolizumab is a humanized anti–programmed cell death 1 (PD-1) monoclonal antibody that inhibits PD-1 from binding to programmed cell death-1 ligand-1 (PD-L1). In a phase I clinical trial (KEYNOTE-001), pembrolizumab showed antitumor efficacy for patients with advanced NSCLC and PD-L1 positive expression [1]. This trial concluded that pembrolizumab was more effective in tumor cells with more than 50% PD-L1 expression.

A phase III clinical trial (KEYNOTE-024) enrolled 305 previously untreated patients who were diagnosed with advanced NSCLC, with more than 50% PD-L1 expression. This study revealed that pembrolizumab was more effective in progression-free survival, overall survival, and a higher response rate than platinum-based chemotherapy [4].

From these study results, approval of 22C3 assay was granted by the U.S. food and drug administration as a companion diagnostic to predict the clinical response to pembrolizumab treatment [6–9].

In Japan, small biopsy samples collected by bronchoscopic examination are often used for diagnosing lung cancer [10–13]. However, since surgically resected specimens and core-needle biopsy samples were used to estimate drug potency in past clinical trials, there is little known regarding the reliability of small biopsy samples [14, 15]. In addition, although some studies evaluated the reliability of small biopsy samples, most of these studies were retrospective in nature, or were assessed by different antibodies [13–15].

## Methods

### Aim and study design

The aim of this study is to prospectively investigate the reliability of small samples for NSCLC cases to determine the status of PD-L1 expression. In pathologically confirmed NSCLC, PD-L1 expression was evaluated using companion diagnostic PD-L1 immunohistochemistry at our institution. We evaluated: 1) tumor cell count and sample size, 2) tumor proportion score (TPS): <1, 1–49%, 50%≦ and 3) the concordance rate of TPS by biopsy and surgical samples.

### Patient selection

We prospectively enrolled patients who underwent diagnostic biopsy procedures from March 2017 to August 2018. In this study, patients were examined through Japan’s health insurance, and written informed consent was obtained from all participants. Included patients were suspected of lung cell carcinoma by computed tomography (CT) or positron emission tomography - computed tomography (PET-CT) imaging. Diagnostic biopsy samples were obtained by flexible bronchoscopy and core-needle biopsy, at initial examination.

### Diagnostic procedures

We selected the most appropriate diagnostic method for each case by taking into consideration patient safety and diagnostic rates. For bronchoscopic examinations, endobronchial ultrasonography (EBUS) (Endoscopic Ultrasound Center; EU-ME2, Olympus, Tokyo, Japan) was routinely used in combination with endobronchial ultrasonography guided transbronchial biopsy (EBUS-TBB) and endobronchial ultrasonography guided transbronchial needle aspiration (EBUS-TBNA) with flexible bronchoscope. EBUS-TBB was undertaken using a thin bronchoscope (BF-P260F, Olympus, Tokyo, Japan) or normal bronchoscope (BF-1 T260, Olympus, Tokyo, Japan). For EBUS-TBB, imaging of the peripheral pulmonary lesions was confirmed using a miniature ultrasound probe (UM-S20-17S, 20 MHz center frequency, radial type, Olympus, Tokyo, Japan), and samples were obtained by a guide sheath kit (K-201, 203 guide-sheath kit, Olympus, Tokyo, Japan). After confirmation of the ultrasound probe within the target lesion, brushing and biopsy forceps were performed alternately, for a minimum of 5 times. EBUS-TBNA was undertaken using a flexible fiberscope (BF-UC260F, Olympus, Tokyo, Japan), and performed 2 to 3 times with a 22-gauge needle (Single Use Aspiration Needle; NA-201SX-4022, Olympus, Tokyo, Japan). CT-guided core needle biopsy was performed 2 to 3 times with a semi-automatic aspiration device (Temno Evolution, Care Fusion Japan, Tokyo, Japan). The needle size was 20 gauge, and the length of the needle was 11 or 15 cm. US-guided core needle biopsy was usually performed at least 3 times.

### Pathological diagnosis

Pathological diagnosis was conducted by pathologist using hematoxylin-eosin (HE) stained slides. After the diagnosis of NSCLC, PD-L1 immunohistochemistry staining of each biopsy sample was conducted and assessed by at least two pathologists.

**Fig. 1:**
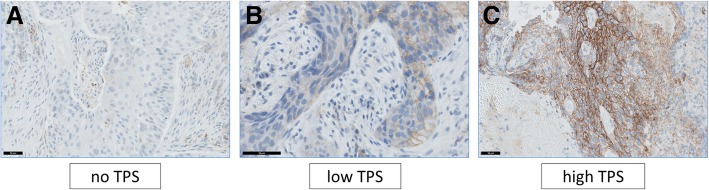
The typical cases for each TPS level: < 1, 1–49%, 50%≦. **a** The TPS was under 1%; no TPS. **b** The TPS was 20%; low TPS. **c** The TPS was 80%; high TPS. The TPS level was evaluated by pathologists who completed training courses in TPS estimation TPS: tumor proportion score

The samples, which were formalin-fixed and paraffin-embedded, were sliced at a thickness of 4 μm. The sections were processed for 20 min at 97 °C for deparaffinized and inactivating enzymes. Sequentially, the samples were stained for PD-L1 with an anti-human PD-L1 antibody. PD-L1 expression was evaluated in our institution using companion diagnostic PD-L1 immunohistochemistry (IHC) (PD-L1 IHC 22C3, pharmDx, Dako/Agilent, Santa Clara, United States) with autostainer Link 48, detecting driver mutation in parallel. Following the standard recommendation of previous publications, PD-L1 protein expression was determined by TPS, which is the percentage of viable tumor cells showing partial or complete membrane staining. PD-L1 expression was classified into three levels: no TPS (<1%), low TPS (1–49%), and high TPS (50%≦). Figure 1 shows typical cases for each TPS level. Using HE and PD-L1 stained slides, we manually assessed the number of tumor cells, the sample size (diameter), the crush rate with a cut-off value of <5, 5–50%, 50%<, and the TPS for each biopsy sample using the slide that contained the most tumor cells.

### Comparison of small biopsy samples and surgical specimens

In patients assessed as early stage NCSLC by diagnostic procedures, small biopsy and resected specimens were compared from the standpoint of TPS after surgical operation. We evaluated the concordance rate for TPS, which was under the 20% threshold to differentiate between small biopsy and surgical specimen.

### Statistical analysis

JMP pro 13 (SAS Institute Japan, Tokyo, Japan) was used for statistical analysis. We analyzed the differences of tumor cell counts and sample sizes in each method non-parametrically using the Wilcoxon rank-sum test. A *P*-value of less than 0.05 was considered statistically significant.

## Results

A total of 153 patients were eligible for this study. Patients’ characteristics are shown in Table 1. The biopsy methods performed for each case were: 110 for EBUS-TBB; 84 for thin bronchoscope, 26 for normal bronchoscope; 23 for EBUS-TBNA, and 20 cases were core-needle biopsy. For pathological subtypes, 59.5% were adenocarcinoma, 35.9% were squamous cell carcinoma, and 4.6% were others. Smoking histories were observed for 86.3% of patients.

**Table 1:** Characteristics of Patients

	Biopsy Methods			
	TBB (BF: P260F)	TBB (BF: 1 T260)	TBNA	Core-needle (CT or US)
Patients	84	26	23	20
Age				
Mean	73.4	68.6	68.3	65.9
Range	49–88	45–84	48–88	42–93
Sex				
Male	56	20	16	14
Female	28	6	7	6
Smoking status				
Current/Ex	70	23	21	18
Never	14	3	2	2
Pathological subtypes				
Adeno	59	7	13	12
Squamous	23	18	9	5
Others	2	1	1	3

*TBB* transbronchial biopsy, *TBNA* transbronchial needle aspiration, *Adeno* adenocarcinoma, Squamous: squamous cell carcinoma

Table 2 shows the number of tumor cells, crush artifact or necrotic changes, and sample sizes of each specimen and for each method. Ninety-three percent of cases contained enough tumor cells (over 100 tumor cells) for TPS evaluation. Normal size bronchoscope biopsy method was able to obtain more tumor cells than thin bronchoscope. In small samples that were obtained by thin bronchoscopy, 35.7% of cases showed a crush artifact rate of more than 50%. In this study, the sample sizes for normal bronchoscope and core-needle were significantly larger compared with other methods (Table 3).

**Table 2:** Tumor cell counts, crush artifact or necrotic changes, and sample size for each method

	TBB (BF: P260F)	TBB (BF: 1 T260)	TBNA	Core-needle (CT or US)
Sample number	84	26	23	20
Tumor cell counts				
<100	11	0	0	0
100≦, <1000	56	3	14	3
1000≦, <2000	14	3	0	5
2000<	3	20	9	12
Median	559	6953	1968	6593
Range	30–3000	380–30,000	100–9000	100–37,500
Crush artifact or necrotic change, %				
<5	15	17	12	14
5–50	39	6	8	4
50<	30	3	3	2
Diameter of biopsy sample, mm, mean (95% C.I.)				
Major axis	1.3 (±0.1)	3.2 (±0.9)	1.6 (±0.4)	6.3 (±1.7)
Minor axis	1.0 (±0.1)	2.2 (±0.6)	1.3 (±0.4)	1.1 (±0.8)

*TBB* transbronchial biopsy, *TBNA* transbronchial needle aspiration

**Table 3:** *P*-value for each method

	*P*-value	
	tumor cell count	sample size
BF P260F vs 1 T260	*p* < 0.0001	*p* < 0.0001
BF P260F vs TBNA	*p* = 0.0128	*p* = 0.2317
BF P260F vs core needle	*p* < 0.0001	*p* < 0.0001
BF 1 T260 vs TBNA	*p* = 0.0004	*p* < 0.0001
BF 1 T260 vs core needle	*p* = 0.7229	*p* = 0.0027
TBNA vs core needle	*p* = 0.0057	*p* < 0.0001

*TBNA* transbronchial needle aspiration

**Fig. 2:**
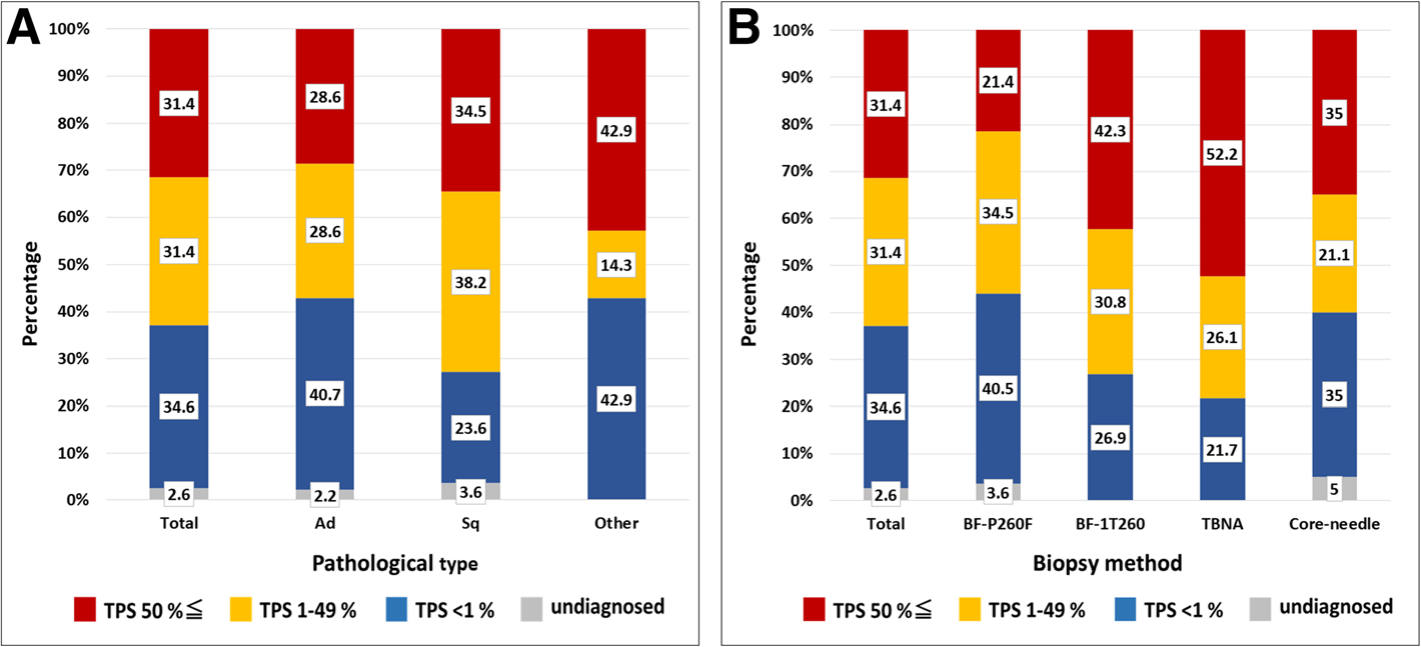
**a** The total percentage of TPS for each pathological case. **b** The total percentage of TPS for each biopsy method. Red indicates high TPS (50%≦), yellow indicates low TPS (1–49%), and blue indicates no TPS (<1%). Gray indicates undiagnosed cases. Ad: adenocarcinoma, Sq: squamous cell carcinoma. TBNA: transbronchial needle aspiration

Figure 2a shows the percentage of TPS for all pathological cases. The ratio of high TPS was 31.4%, low TPS was 31.4% and no TPS was 34.6%. While squamous cell carcinoma represented 72.7% of TPS positive cases, adenocarcinoma was comprised of 57.2%. For each method, TPS positive cases using thin bronchoscope was 55.9%, normal bronchoscope was 73.1%, and EBUS-TBNA was 78.3% (Fig. 2b).

Table 4 shows TPS expression by cancer stage. In adenocarcinoma, early stage cases (stageI and II), showed 16.1% high TPS compared to advanced stage cases (stage III and IV), with 34.5%. In early stage cases, 48.4% showed no TPS. In squamous cell carcinoma, the rates of TPS were similar regardless of stage.

**Table 4:** The difference of TPS by staging

	TPS			
	high	low	no	undiagnosed
Adenocarcinoma				
StageI, II	5	11	15	0
(%)	16.1	35.5	48.4	0
StageIII, IV	20	15	21	2
(%)	34.5	25.9	36.2	3.5
Squamous cell carcinoma				
StageI, II	6	6	4	1
(%)	35.3	35.3	23.5	5.9
StageIII, IV	12	14	7	1
(%)	35.3	41.2	20.6	2.9

**Fig. 3:**
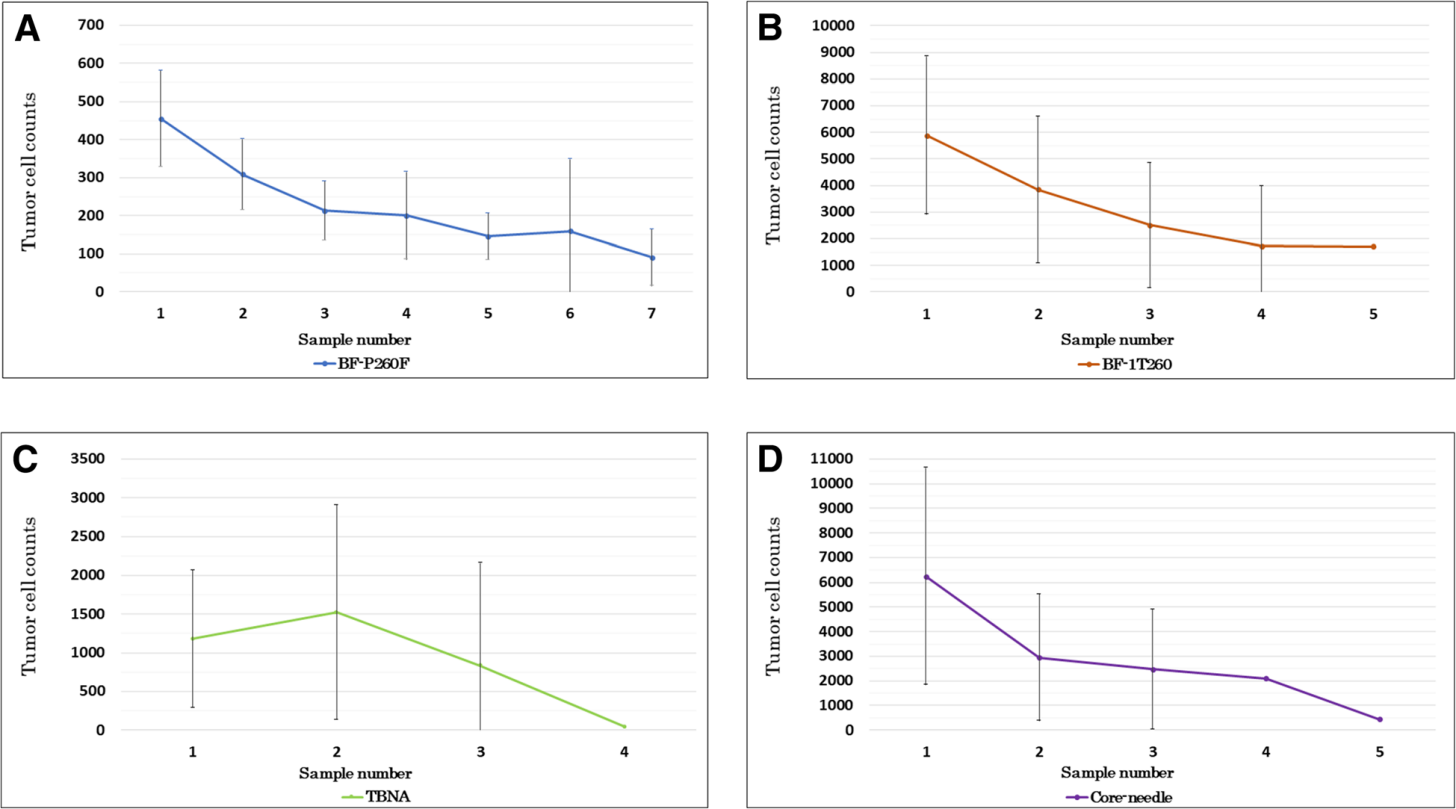
The subsequent median number of tumor cell counts with 95% confidential interval for each biopsy method. **a** Comparison of tumor cells using thin bronchoscopy (BF-P260F), (**b**) normal bronchoscopy (BF-1 T260), (**c**) EBUS-TBNA, and (**d**) CT or US-guided core-needle biopsy. EBUS-TBNA: endobronchial ultrasonography - transbronchial needle aspiration. CT: computed-tomography. US: ultra-sonography

Tumor cell counts in biopsy samples for each method are shown in Fig. 3. We were able to obtain over 100 tumor cells in nearly every case, which is necessary for evaluating PD-L1 expression. These results showed that earlier samples obtained more tumor cells. However, after the first biopsy, the number of tumor cells for subsequent biopsies decreased. In 11 cases, there were less than 100 tumor cells per sample.

Table 5 shows a comparison of PD-L1 expression between smaller biopsy samples and surgical specimens in 30 cases. Smaller biopsies were taken by thin bronchoscope. The concordance rate was 86.7%, which was under the 20% threshold to differentiate TPS between small biopsy and resected samples. Additional file 1 shows all raw data of this study.

**Table 5:** The comparison of PD-L1 expression between small biopsy samples and surgical specimens

Case	Sex	Smoking	Location	Pathology (dominant %)	Size of lesion (mm)	SUV max	BF	Tumor cell count for BF	TPS in BF (%)	TPS in operation (%)
1	F	never	Ling	Ad (papi 85)	24 × 20	4.0	P260F	700	0	10
2	M	former	RL	Ad (acinar 70)	17 × 13	4.0	P260F	160	0	0
3	M	former	Ling	Ad (enteric)	18 × 18	5.6	P260F	600	0	10
4	M	current	LL	Pleomorphic	40 × 28	8.1	P260F	500	50	100
5	M	current	RL	Sq	40 × 35	13.4	P260F	400	20	30
6	F	never	LU	Ad (lepidic 90)	17 × 16	4.1	P260F	140	70	70
7	F	former	LU	Ad (papi 70)	25 × 22	4.6	P260F	1200	0	10
8	M	former	RU	Ad (lepi/aci 60/30)	27 × 25	20.4	P260F	800	0	0
9	F	never	LU	Ad (solid/lepi 60/30)	25 × 18	9.4	P260F	70	50	0
10	F	never	LL	Ad (lepi/aci/papi 50/20/20)	25 × 25	3.9	P260F	280	0	0
11	M	former	LL	Ad (papi 60)	18 × 16	6.3	P260F	220	50	30
12	F	former	RU	Ad (papi/lepi 90/10)	15 × 12	3.1	P260F	170	0	0
13	M	former	LL	Ad (solid 100)	43 × 40	11	P260F	160	90	100
14	F	never	LU	Ad (aci/lepi/papi/micropapi 40/20/20/20)	18 × 17	2.2	P260F	120	40	10
15	M	former	RU	Ad (papi/lepi/solid 30/10/10)	27 × 24	2.9	P260F	340	20	10
16	M	former	LU	Ad (solid//papi/aci 50/30/20)	26 × 12	3.2	P260F	220	0	0
17	M	former	RU	Ad (aci/lepi/papi 65/30/5)	36 × 20	5.3	P260F	530	30	30
18	M	former	RL	Mucinous	64 × 29	9.3	P260F	450	0	0
19	M	former	LU	Squamous	20 × 15	8	P260F	450	30	10
20	F	former	RU	Squamous	12 × 8	14.2	P260F	1000	0	0
21	M	never	RU	Ad (papi/aci/lepi/micropapi 60/20/10/10)	33 × 20	3.4	P260F	100	0	10
22	M	former	RU	Ad (papi/lepi 60/40)	35 × 25	6	P260F	100	30	0
23	F	former	RM	Ad (papi/lepi/aci 60/20/20)	23 × 18	3.8	P260F	700	0	10
24	M	former	RU	Ad (micropapi/aci/papi/lepi 50/20/20/10)	13 × 13	3.1	P260F	600	10	20
25	M	former	LU	Mucinous	25 × 23	1.7	P260F	200	10	0
26	M	former	RM	Large	30 × 25	4.8	P260F	200	70	90
27	M	former	RU	Mucinous	70 × 35	8.2	P260F	300	0	0
28	M	former	RU	Squamous	45 × 20	7	P260F	350	0	0
29	F	former	RU	Ad (solid/papi 80/20)	45 × 38	14.1	P260F	1000	20	0
30	F	former	LU	Squamous	50 × 30	4.8	P260F	50	0	10

*Aci* acinar, *Papi* papillary, *lepi* lepidic, *micropapi* micropapillary, *SUV* standardized uptake value, *RU* right upper lobe, *RM* right middle lobe, *RL* right lower lobe, *LU* left upper segment, *Ling* left lingular segment, *LL* left lower lobe

## Discussion

This is the first report, to our knowledge, to prospectively investigate TPS for small biopsy samples in clinical practice. Bronchoscopic examinations are widely conducted as an initial diagnostic procedure. Hence, the assessment of reliability for small samples is important in the decision-making process for induction of immuno-checkpoint inhibitor as a first line treatment [1, 4, 5, 13–15].

For each case in this study, the ratio of TPS (<1, 1–49%, 50%≦) was approximately equal to past studies [5]. In a previous report outlining TPS assessment guidelines, samples should contain at least 100 tumor cells for TPS assessment [16]. Although there were differences in the sample sizes for each method, we were able to obtain at least 100 tumor cells for our small samples. In particular, this study revealed the differences in specimen size between thin bronchoscopy, normal bronchoscopy and other methods. Moreover, our approach revealed that tumor cells from subsequent biopsies decreased which might be due to localized bleeding from repeated biopsies. Therefore, the first and second biopsy samples are considered important for the evaluation of TPS. When there were less than 100 tumor cells per sample, we collected subsequent samples and combined these on one slide for TPS evaluation.

For each pathological subtype, the ratio of TPS positive cases was higher in squamous cell carcinoma as previously reported [17]. These cases were more likely located in the central airway and therefore, normal bronchoscopy could easily reach the target lesion and collect an appropriate specimen size.

For adenocarcinoma cases, there were some differences observed for TPS between early and advanced cancer stages. It has been suggested that PD-L1 expression increases as the stage of cancer advances. On the other hand, in squamous cell carcinoma, there were no significant differences seen for TPS between early and advanced stage cases. Differences in the pathological diagnosis and cancer staging might influence PD-L1 expression [17–19].

The samples that were obtained by thin bronchoscope tended to collect less tumor cells and show low TPS. This was mainly due to early stage adenocarcinoma, located at the pulmonary peripheral areas, which tended to show low TPS [20]. Another explanation might be that the crush artifact rates were relatively higher for thin bronchoscope samples as previously reported [13]. However, for the comparison of bronchoscopic specimens, which were obtained by thin bronchoscopy or surgically resected, the TPS concordance was relatively high which contrasts with previous retrospective reports [14, 15].

There were some limitations observed in this study. First, this study was performed at a single institution; therefore, these results should be compared against larger multi-center studies. Second, there were less cases for normal bronchoscope, EBUS-TBNA, and core-needle in compared to the number of cases using thin bronchoscope. However, we believe this study emulates real-world circumstances since there are more instances to perform thin bronchoscope. Third, this study included eight post chemotherapy patients (re-biopsy) and seven recurrences after surgery. It could be suggested that these treatments may have influenced PD-L1 expression as previous papers have reported [21]. Forth, this study focused on TPS data only; however, we plan to further evaluate the response of other immune checkpoint inhibitors in future studies.

## Conclusion

Small biopsy samples obtained by bronchoscopy were deemed appropriate to evaluate TPS, and the frequency of TPS was comparable to past clinical trials using larger samples for evaluation. Differences in TPS were observed according to diagnostic tools, cancer histologic types and staging. The TPS concordance rate between EBUS-TBB samples and surgical materials was high.

## Additional file

Additional file 1: The raw data of all cases in this study. (PDF 252 kb)

## Data Availability

All data generated or analyzed during this study are included in this published article and its Additional file 1.

## Abbreviations

EBUS: Endobronchial ultrasonography
EBUS-TBB: Endobronchial ultrasonography - transbronchial biopsy
EBUS-TBNA: Endobronchial ultrasonography - transbronchial needle aspiration
NSCLC: Non-small cell lung cancer
PD-1: Programmed cell death 1
PD-L1: Programmed cell death-1 ligand-1
TPS: Tumor proportion score
