# Supplementary material for: A prospective observational study to assess PD-L1 expression in small biopsy samples for non-small-cell lung cancer
Source: BMC Cancer. 2019 Jun 7;19:546. doi: 10.1186/s12885-019-5773-3 (PMC6555021; doi:10.1186/s12885-019-5773-3)
Supplement: Supplementary file 1 — The raw data of all cases in this study. (PDF 252 kb) [file 12885_2019_5773_MOESM1_ESM.pdf]

Patient	Smoke	Method	Pathology	Stage	Tumor cell	TPS
P1	Ex	P260F	Sq	1	150≦	80
P2	C	P260F	Sq	4	150≦	＜1
P3	Ex	P260F	Sq	2	150≦	20
P4	Ex	P260F	NSCLC	1	150≦	90
P5	Ex	P260F	Ad	2	150≦	＜1
P6	C	P260F	Ad	4	150≦	＜1
P7	Ex	P260F	Ad	2	150≦	10
P8	N	P260F	Ad	4	150≦	90
P9	Ex	P260F	Ad	4	150≦	＜1
P10	N	P260F	Ad	4	＜100	＜1
P11	Ex	P260F	Sq	3	150≦	10
P12	Ex	P260F	Sq	4	150≦	30
P13	N	P260F	Ad	4	150≦	＜1
P14	Ex	P260F	Ad	4	150≦	10
P15	N	P260F	Sq	2	150≦	80
P16	N	P260F	Ad	4	150≦	10
P17	N	P260F	Ad	4	150≦	＜1
P18	Ex	P260F	Sq	4	150≦	10
P19	Ex	P260F	Sq	3	150≦	＜1
P20	N	P260F	Ad	4	100-150	undiagnosed
P21	Ex	P260F	Sq	4	150≦	30
P22	Ex	P260F	LCNEC	4	150≦	＜1
P23	C	P260F	Ad	4	150≦	90
P24	Ex	P260F	Ad	4	＜100	＜1
P25	C	P260F	Sq	2	150≦	undiagnosed
P26	Ex	P260F	Sq	4	150≦	80
P27	Ex	P260F	Sq	1	150≦	30
P28	Ex	P260F	Ad	2	150≦	＜1
P29	Ex	P260F	Ad	4	150≦	＜1
P30	Ex	P260F	Ad	4	150≦	10
P31	N	P260F	AD	4	150≦	80
P32	Ex	P260F	Ad	4	＜100	90
P33	Ex	P260F	Sq	1	150≦	＜1
P34	N	P260F	Ad	4	150≦	＜1
P35	C	P260F	Ad	4	＜100	undiagnosed
P36	Ex	P260F	Sq	1	150≦	＜1
P37	Ex	P260F	Ad	4	150≦	40
P38	Ex	P260F	Ad	1	＜100	＜1
P39	Ex	P260F	Sq	4	150≦	60
P40	C	P260F	Sq	3	150≦	2
P41	Ex	P260F	Ad	4	100-150	10
P42	N	P260F	Ad	1	150≦	＜1
P43	Ex	P260F	Ad	1	150≦	＜1
P44	C	P260F	Ad	1	150≦	＜1
P45	Ex	P260F	Ad	3	150≦	50
P46	Ex	P260F	Sq	2	150≦	20
P47	N	P260F	Ad	1	100-150	70
P48	C	P260F	Ad	3	150≦	＜1
P49	Ex	P260F	Ad	1	150≦	＜1
P50	N	P260F	Ad	1	＜100	50
P51	Ex	P260F	Ad	1	150≦	＜1
P52	C	P260F	Ad	1	150≦	50
P53	N	P260F	Ad	1	150≦	＜1
P54	Ex	P260F	Ad	3	150≦	90
P55	C	P260F	Ad	1	150≦	40
P56	C	P260F	Ad	1	150≦	20

P57	C	P260F	Ad	1	150≦	＜1
P58	Ex	P260F	Ad	1	150≦	30
P59	Ex	P260F	Ad	1	150≦	＜1
P60	Ex	P260F	Sq	3	150≦	＜1
P61	Ex	P260F	Ad	1	150≦	70
P62	Ex	P260F	Ad	1	150≦	10
P63	C	P260F	Small/Sq	1	150≦	30
P64	Ex	P260F	Ad	1	150≦	10
P65	Ex	P260F	Ad	2	150≦	＜1
P66	Ex	P260F	Ad	4	150≦	＜1
P67	C	P260F	Ad	4	150≦	＜1
P68	Ex	P260F	Ad	2	150≦	20
P69	Ex	P260F	Ad	4	150≦	5
P70	N	P260F	Ad	1	150≦	10
P71	N	P260F	Ad	1	150≦	＜1
P72	N	P260F	Ad	1	150≦	＜1
P73	Ex	P260F	Ad	1	150≦	30
P74	N	P260F	Ad	1	150≦	＜1
P75	C	P260F	Ad	4	150≦	30
P76	Ex	P260F	Ad	1	150≦	90
P77	C	P260F	Sq	1	150≦	70
P78	C	P260F	Sq	4	150≦	10
P79	Ex	P260F	Ad	4	150≦	10
P80	C	P260F	Ad	4	150≦	10
P81	Ex	P260F	Sq	3	150≦	30
P82	C	P260F	Sq	1	150≦	＜1
P83	Ex	P260F	Ad	4	＜100	90
P84	Ex	P260F	Ad	4	150≦	＜1

Age
49-88

Amount of tumor cell							
Patient	Sample 1	Sample 2	Sample 3	Sample 4	Sample 5	Sample 6	Sample 7
P1	1500	100	230				
P2	230	250	350	150	150		
P3	150	250	300	250			
P4	800						
P5	700						
P6	200	150	50				
P7	600	600					
P8	1500	600					
P9	700	1800	150				
P10	30						
P11	1100	400					
P12	1100	700	800				
P13	1400						
P14	300	15	170				
P15	1000	350					
P16	2800	200	500	160	20		
P17	58	26	42				
P18	900	650	20				
P19	1820	1820					
P20	100	130	13				
P21	20	900					
P22	1000						
P23	200	120	20	5	50		
P24	30	30	50	30			
P25	170						
P26	100	40	40	160	350		
P27	70	170	450	440			
P28	550	40	30	130	350	130	
P29	900	700	420	90	120	20	120
P30	40	70	16	0	11	15	
P31	320	60	480	150	240	80	90
P32	100	12	30	10			
P33	500	40					
P34	7	30	90	40	10	70	
P35	100	10	20	40			
P36	150	1000	800	900			
P37	100	130	40	70	80	600	60
P38	3	40					
P39	780						
P40	140	920	160				
P41	140						
P42	700	50					
P43	160	140					
P44	200	250					
P45	3000	300	150				
P46	400						
P47	140						
P48	700	1200					
P49	45	800					
P50	70	70					
P51	280	160					
P52	200	220					
P53	70	170					
P54	160	130					
P55	40	120					

P56	340						
P57	220						
P58	470	530					
P59	350	450					
P60	500						
P61	500	700	200	150			
P62	50	100					
P63	1000	200					
P64	50						
P65	300	200					
P66	100	50	100	300	200		
P67	50	20	30				
P68	300	100	150	30	100		
P69	200	200	200				
P70	200						
P71	40	80	30	50	200	200	
P72	100	50					
P73	200	200	100	50			
P74	300	100	300				
P75	1000	500	800	400	100		
P76	100	300					
P77	2000						
P78	100	300					
P79	100	150	30				
P80	100	30					
P81	500	80					
P82	400	300	500	1000	200		
P83	30						
P84	30	50					
AVE	454.7976	308.2537	212.4595	200.2174	145.4	159.2857	90
95%CI	581.2476	401.5763	290.2715	315.1036	206.76446	348.5297	164.5241
	328.3477	214.9311	134.6475	85.33115	84.035536	-29.9582	15.47587

Patient	Crush rate	MajorAxis	MinorAxis
P1	2	50	30
P2	2	70	50
P3	2	60	40
P4	1	25	15
P5	1	20	20
P6	3	50	50
P7	3	60	40
P8	3	140	40
P9	2	80	40
P10	1	5	5
P11	3	70	40
P12	2	70	30
P13	1	30	30
P14	3	20	20
P15	3	50	50
P16	2	90	60
P17	3	30	30
P18	2	40	40
P19	1	100	50
P20	3	30	10
P21	3	50	40
P22	2	90	30
P23	2	30	50
P24	3	40	50
P25	3	30	40
P26	2	50	40
P27	3	30	20
P28	2	70	50
P29	3	30	30
P30	3	40	40
P31	2	60	50
P32	2	50	30
P33	2	40	40
P34	3	50	30
P35	3	50	40
P36	2	50	40
P37	2	50	50
P38	2	50	40
P39	1	30	30
P40	2	40	40
P41	3	50	40
P42	1	60	40
P43	2	40	30
P44	2	50	30
P45	1	100	50
P46	1	30	30
P47	2	40	40
P48	1	70	50
P49	2	40	40
P50	3	70	20
P51	2	80	50
P52	2	60	40
P53	3	40	60
P54	2	20	50
P55	2	50	50
P56	2	20	20

P57	2	100	50
P58	1	70	50
P59	1	50	50
P60	2	100	100
P61	3	60	60
P62	3	40	30
P63	1	50	50
P64	3	50	30
P65	2	50	50
P66	2	40	30
P67	3	30	30
P68	2	50	40
P69	3	50	50
P70	2	40	40
P71	2	50	50
P72	3	40	40
P73	3	30	40
P74	2	70	60
P75	2	50	50
P76	3	70	40
P77	2	120	60
P78	3	50	40
P79	2	60	50
P80	3	50	30
P81	1	70	50
P82	1	60	60
P83	3	30	30
P84	2	40	30
	2.178571	52.5 x25μm	40.47619 x25μm
		1.313 mm	1.012 mm
95%CI		1.438 mm	1.085 mm
		1.187 mm	0.94 mm

Crush rate	
＜5%	1
5-50%	2
≦50%	3

Patient	Smoke	Method	Pathology	Stage	Tumor cell	TPS
1T-1	C	1T	Sq	3	150≦	70
1T-2	C	1T	Ad	3	150≦	＜1
1T-3	C	1T	Sq	4	150≦	50
1T-4	Ex	1T	Sq	4	150≦	＜1
1T-5	C	1T	Sq	3	150≦	60
1T-6	Ex	1T	Ad	3	150≦	50
1T-7	C	1T	Ad	3	150≦	＜1
1T-8	N	1T	Sq	3	150≦	＜1
1T-9	Ex	1T	Sq	3	150≦	70
1T-10	N	1T	Sq	4	150≦	10
1T-11	C	1T	Ad	3	150≦	100
1T-12	C	1T	Sq	1	150≦	40
1T-13	Ex	1T	Ad	4	150≦	60
1T-14	Ex	1T	Sq	4	150≦	20
1T-15	Ex	1T	Sq	3	150≦	80
1T-16	Ex	1T	Sq	3	150≦	＜1
1T-17	N	1T	Ad	4	150≦	＜1
1T-18	Ex	1T	Ad	1	150≦	10
1T-19	C	1T	Sq	4	150≦	20
1T-20	Ex	1T	Sq	redeveloped	150≦	＜1
1T-21	Ex	1T	Sq	2	150≦	90
1T-22	Ex	1T	Sq	2	150≦	70
1T-23	Ex	1T	Sq	redeveloped	150≦	50
1T-24	Ex	1T	NSCLC	3	150≦	10
1T-25	C	1T	Sq	2	150≦	20
1T-26	C	1T	Sq	4	150≦	10

Age
45-84

Amount of tumor cell					
Patient	Sample 1	Sample 2	Sample 3	Sample 4	Sample 5
1T-1	14500				
1T-2	5600	2100			
1T-3	5000	3200			
1T-4	2400	5600			
1T-5	1400				
1T-6	2000	2300			
1T-7	1300	400	2700		
1T-8	70	3400	900		
1T-9	11000	5500	5500	4500	
1T-10	16000	24000			
1T-11	10600				
1T-12	4600	6800	13000	4500	1700
1T-13	3300				
1T-14	3100	600	2100	400	
1T-15	3100	1300	400		
1T-16	2700	50	3400	400	
1T-17	60	800	70	10	
1T-18	100	380	80		
1T-19	21000	2200	200	550	
1T-20	30000	15000			
1T-21	1000	700	700		
1T-22	7000				
1T-23	5000				
1T-24	1000	2000			
1T-25	600	100			
1T-26	300	500	1000		
AVE	5874.231	3846.5	2504.167	1726.667	1700
95%CI	8840.403	6598.659	4848.634	3988.892	
	2908.058	1094.341	159.6996	-535.559	

Patient	Crush rate
1T-1	1
1T-2	1
1T-3	1
1T-4	2
1T-5	2
1T-6	1
1T-7	1
1T-8	1
1T-9	1
1T-10	1
1T-11	1
1T-12	1
1T-13	1
1T-14	2
1T-15	1
1T-16	1
1T-17	3
1T-18	2
1T-19	1
1T-20	1
1T-21	1
1T-22	1
1T-23	2
1T-24	3
1T-25	3
1T-26	2
	1.4615385

MajorAxis	
200	
220	
100	
100	
100	
75	
80	
90	
200	
270	
400	
120	
90	
90	
100	
130	
40	
50	
100	
300	
100	
250	
150	
80	
100	
90	
139.4231	x25μm
3.486 mm	

4.359 mm
2.612 mm

MinorAxis	
130	
130	
60	
60	
40	
50	
60	
60	
140	
180	
300	
100	
40	
60	
60	
100	
30	
30	
100	
100	
80	
100	
100	
60	
80	
40	
88.07692	x25μm
2.202 mm	

2.777 mm
1.626 mm

Crush rate	
＜5%	1
5-50%	2
≦50%	3

Patient	Smoke	Method	Pathology	Stage	Tumor cell	TPS	
NA1	Ex	TBNA	Ad	3	150≦	90	
NA2	Ex	TBNA	Sq	4	150≦	30	
NA3	Ex	TBNA	meta Ca	2	150≦	＜1	
NA4	Ex	TBNA	Sq	redeveloped	150≦	10	
NA5	N	TBNA	Ad	3	150≦	30	
NA6	Ex	TBNA	Sq	4	150≦	60	
NA7	Ex	TBNA	Sq	4	150≦	80	
NA8	Ex	TBNA	Sq	2	100-150	＜1	
NA9	Ex	TBNA	Ad	4	150≦	＜1	
NA10	Ex	TBNA	Ad	3	150≦	90	
NA11	N	TBNA	Ad	4	150≦	10	
NA12	Ex	TBNA	Ad	4	150≦	80	
NA13	Ex	TBNA	Sq	4	100-150	＜1	
NA14	C	TBNA	Ad	4	150≦	10	
NA15	Ex	TBNA	Ad	redeveloped	150≦	＜1	
NA16	C	TBNA	Ad	3	150≦	50	
NA17	Ex	TBNA	Ad	4	150≦	100	
NA18	C	TBNA	Sq	3	150≦	100	
NA19	C	TBNA	Ad	3	150≦	90	
NA20	Ex	TBNA	Sq	3	150≦	60	
NA21	Ex	TBNA	Sq	4	150≦	40	
NA22	C	TBNA	Ad	redeveloped	150≦	80	
NA23	Ex	TBNA	Ad	4	150≦	60	

Age
48-88

	Amount of tumor cell				
	Patient	Sample 1	Sample 2	Sample 3	Sample 4
	NA1	3600			
	NA2	500	300	600	
	NA3	330			
	NA4	600	400		
	NA5	88	630		
	NA6	200			
	NA7	230	500	33	
	NA8	100			
	NA9	3800			
	NA10	3000	33		
	NA11	9000	3500		
	NA12	1300	9000		
	NA13	100	40		
	NA14	2200	550		
	NA15	80	6300	700	40
	NA16	100	100		
	NA17	100	300		
	NA18	200			
	NA19	800	300	2000	
	NA20	100	2000		
	NA21	200			
	NA22	200	150		
	NA23	300	200		
	AVE	1179.478	1518.938	833.25	40
	95%CI	2071.719	2904.925	2156.253	
		287.238	132.9499	−489.753	

Patient	Crush rate	MajorAxis	MinorAxis
NA1	2	100	60
NA2	2	30	20
NA3	2	50	20
NA4	2	30	10
NA5	1	60	45
NA6	3	150	100
NA7	3	50	50
NA8	1	10	10
NA9	1	80	50
NA10	1	80	50
NA11	1	100	70
NA12	1	100	50
NA13	1	10	10
NA14	1	70	70
NA15	1	50	50
NA16	2	50	50
NA17	2	100	100
NA18	1	30	50
NA19	1	150	150
NA20	1	70	70
NA21	2	20	20
NA22	2	50	50
NA23	3	40	30
	1.6086957	64.34783 x25μm	51.52174 x25μm
		1.609 mm	1.288 mm

	MajorAxis	MinorAxis
95%CI	2.03 mm	1.646 mm
	1.188 mm	0.93 mm

Crush rate	
＜5%	1
5–50%	2
≦50%	3

Patient	Smoke	Method	Pathology	Stage	Tumor cell	TPS	
CT1	Ex	Core-needle	Ad	3	150≦	90	
CT2	C	Core-needle	Carcinoma	4	150≦	80	
CT3	Ex	Core-needle	Sq	2	150≦	90	
CT4	C	Core-needle	Sq	redeveloped	150≦	＜1	
CT5	Ex	Core-needle	Ad	3	150≦	＜1	
CT6	Ex	Core-needle	Sq	4	150≦	undiagnosed	
CT7	Ex	Core-needle	Ad	4	150≦	＜1	
CT8	Ex	Core-needle	MetaCa	2	150≦	70	
CT9	C	Core-needle	Sq	4	150≦	70	
CT10	Ex	Core-needle	Ad	3	150≦	70	
CT11	N	Core-needle	Ad	4	150≦	60	
CT12	Ex	Core-needle	Ad	4	150≦	30	
CT13	Ex	Core-needle	Ad	4	150≦	30	
CT14	C	Core-needle	Ad	4	150≦	30	
CT15	Ex	Core-needle	Ca	redeveloped	150≦	＜1	
CT16	C	Core-needle	Ad	3	150≦	＜1	
CT17	N	Core-needle	Ad	4	150≦	＜1	
CT18	Ex	Core-needle	Sq	4	100-150	30	
CT19	Ex	Core-needle	Ad	3	150≦	＜1	
CT20	Ex	Core-needle	Ad	2	150≦	40	

Age
42-93

Amount of tumor cell					
Patient	Sample 1	Sample 2	Sample 3	Sample 4	Sample 5
CT1	2900				
CT2	500	1000	1900		
CT3	330	180	1000		
CT4	1000	3000			
CT5	4000				
CT6	1400				
CT7	22000				
CT8	37500				
CT9	180	200	360	300	
CT10	10400				
CT11	5200	3900	3500	3900	440
CT12	720	10	1500		
CT13	12000	12000	8000		
CT14	3100	5000			
CT15	15300				
CT16	100				
CT17	1400				
CT18	100				
CT19	2800	2200			
CT20	4000	2000	1000		
AVE	6246.5	2949	2465.714	2100	440
95%CI	10656.35	5518.942	4903.327	24971.17	
	1836.65	379.0584	28.10173	-2077117	

Patient	Crush rate	MajorAxis	MinorAxis
CT1	1	150	15
CT2	1	200	20
CT3	2	450	35
CT4	1	160	25
CT5	1	200	25
CT6	1	70	30
CT7	1	300	25
CT8	1	400	320
CT9	2	40	20
CT10	1	300	50
CT11	1	400	30
CT12	2	120	20
CT13	2	450	40
CT14	1	250	30
CT15	1	300	30
CT16	3	200	30
CT17	1	280	20
CT18	3	130	20
CT19	1	550	50
CT20	1	100	50
	1.4	252.5 x25μm	44.25 x25μm
		6.313 mm	1.106 mm

95%CI	7.965 mm	1.876 mm
	4.66 mm	0.337 mm

Crush rate	
＜5%	1
5–50%	2
≦50%	3
